# Pair barracuda swarm optimization algorithm: a natural-inspired metaheuristic method for high dimensional optimization problems

**DOI:** 10.1038/s41598-023-43748-w

**Published:** 2023-10-25

**Authors:** Jia Guo, Guoyuan Zhou, Ke Yan, Yuji Sato, Yi Di

**Affiliations:** 1 School of Information Engineering, Hubei University of Economics, Wuhan, 430205 China; 2 Hubei Internet Finance Information Engineering Technology Research Center, Wuhan, 430205 China; 3 College of Informatics, Huazhong Agricultural University, Wuhan, 430070 China; 4 China Construction Third Engineering Bureau Installation Engineering Co., Ltd., Wuhan, 43074 China; 5 Faculty of Computer and Information Sciences, Hosei University, Tokyo, 184-8584 Japan

**Keywords:** Engineering, Mathematics and computing, Computational science, Computer science

## Abstract

High-dimensional optimization presents a novel challenge within the realm of intelligent computing, necessitating innovative approaches. When tackling high-dimensional spaces, traditional evolutionary tools often encounter pitfalls, including dimensional catastrophes and a propensity to become trapped in local optima, ultimately compromising result accuracy. To address this issue, we introduce the Pair Barracuda Swarm Optimization (PBSO) algorithm in this paper. PBSO employs a unique strategy for constructing barracuda pairs, effectively mitigating the challenges posed by high dimensionality. Furthermore, we enhance global search capabilities by incorporating a support barracuda alongside the leading barracuda pair. To assess the algorithm’s performance, we conduct experiments utilizing the CEC2017 standard function and compare PBSO against five state-of-the-art natural-inspired optimizers in the control group. Across 29 test functions, PBSO consistently secures top rankings with 9 first-place, 13 second-place, 5 third-place, 1 fourth-place, and 1 fifth-place finishes, yielding an average rank of 2.0345. These empirical findings affirm that PBSO stands as the superior choice among all test algorithms, offering a dependable solution for high-dimensional optimization challenges.

## Introduction

The Particle Swarm Optimization (PSO) algorithm, introduced by Kennedy in 1995^1^, has garnered significant attention from researchers since its inception. It has found successful applications in various practical engineering problems, such as image segmentation^2,3^, sound classification^4^, power planning^5,6^, path planning^7–9^, water pressure control^10^, voltage regulation^11^, sensor networks^12^, among others. Additionally, numerous enhancements have been developed to improve the PSO algorithm. These enhancements typically fall into three main categories: modifying the algorithm’s topology^13^, enhancing the particle swarm learning strategy^14^, and combining PSO with other algorithms.

Changing the topology to design update strategies tailored to particles with distinct characteristics can optimize the utilization of information within the particle swarm^15^. Liang introduced the APSO-C algorithm^16^, which incorporates two key strategies. The first strategy involves partitioning the particle swarm using the k-means method, resulting in subgroups with varying capabilities. The second strategy aims to balance the local and global search aspects of the algorithm. On the other hand, Xu proposed the QLPSO algorithm^17^, which integrates reinforcement learning into the particle swarm algorithm. In this approach, each particle autonomously selects the best topology by referring to a reinforcement learning table that evolves progressively during the iterative process. Comparatively, this experiment demonstrates a faster convergence rate when compared to particle swarm algorithms based on alternative topologies.

Enhancing the learning strategy of the particle swarm algorithm proves to be an effective means of boosting its performance. This improvement can involve adjustments to various learning parameters^18^, such as inertia weights^19^, among others. Tian introduced the MPSO algorithm^20^, which employs a unique approach. It initializes the particle swarm using a logical map and then selects inertia weights using both linear and nonlinear strategies. Furthermore, an auxiliary update mechanism is implemented for global optimal particles, contributing to the algorithm’s robustness. Karim, on the other hand, proposed MPSOEG^21^, an algorithm that optimizes the learning framework by eliminating inertia weights and velocity parameters. Experimental results highlight the algorithm’s efficiency in solving single-objective optimization problems. Wang introduced a novel particle adaptive learning strategy for tackling large-scale optimization problems^22^.

One effective approach to diversify particle swarm information is by combining particle swarm algorithms with other algorithms. Zhu, for instance, integrated the fireworks algorithm with PSO^23^. Dadvar, on the other hand, combined Differential Evolution (DE), a stochastic optimization algorithm, with PSO^24^. This fusion of DE and PSO leverages Nash bargaining theory, demonstrating its superiority over other hybrid models in various applications. In a similar vein, Wang introduced DFS-CPSO^25^, a hybrid algorithm that combines the depth-first search algorithm with the particle swarm algorithm. By integrating the DFS strategy, this approach enhances the diversity of particles and exhibits superior performance, particularly in solving high-dimensional multi-modal problems.

In 2003, Kennedy introduced the bare-bone particle swarm algorithm (BBPSO)^26^, aiming to simplify the PSO by removing intricate parameters. BBPSO employs the Gaussian algorithm during its iterative process, making it more comprehensible. It has been successfully applied to tackle complex problems, such as the traveling salesman problem. Nonetheless, BBPSO is susceptible to getting trapped in local optima^27^. Consequently, numerous researchers have made extensive efforts to enhance the algorithm’s performance in addressing this issue.

In 2014, Campos introduced SMA-BBPSO^28^, an algorithm that employs a matrix following the T distribution to update particle positions. This approach enhances the balance of particles during the iteration process. In 2018, Guo presented DRBBPSO^29^, which incorporates a dynamic reconstruction strategy to bolster the algorithm’s performance by retaining elite particles. This feature helps prevent the algorithm from becoming ensnared in local optima when addressing multi-modal problems. In 2021, Guo proposed CBBPSO^30^, which not only keeps a record of global worst particles but also enhances its capability to solve high-dimensional problems. Subsequently, in 2022, Tian expanded BBPSO by incorporating a transition operator and an orbit merging operator^31^. Then, in 2023, Xiao introduced TMBBPSO^32^, which integrates two memory mechanisms into BBPSO, tailored for solving nonlinear problems. In 2016, Yong introduced the Dolphin Swarm Optimization Algorithm (DSOA)^33^, which simulates the social and hunting behaviors of barracudas within the search area.

Vafashoar introduced two essential mechanisms into BBPSO in their work^34^. Firstly, they employed cellular learning automata (CLA) for parallel computation of mathematical simulation models, facilitating particle flight and path refinement. Secondly, they reoriented particle directions based on the maximum likelihood principle. The combination of these two mechanisms significantly enhances the algorithm’s capability to solve complex optimization problems.

Guo’s FHBBPSO^35^ introduces both a fission and fusion strategy. Initially, the particle swarm is divided into groups using the fission strategy, with each group independently seeking its optimal solution. Subsequently, the fusion strategy is employed to identify the optimal group, followed by another round of fission strategy. This cyclic process continues until the end of the iteration. The fusion strategy draws inspiration from the competitive processes observed in chimpanzee groups, and the combination of these two strategies demonstrates strong performance in solving single-objective optimization problems. Zamani proposed a Quantum-based avian navigation optimizer algorithm^36^ in 2021, a Starling murmuration optimizer^37^ in 2022 Nadimi-Shahraki^38^ proposed an enhanced Moth-Flame optimization method in 2023.

Variants of PSO algorithms find widespread applications in the field of sensors. Kim introduced a novel PSO approach for multi-sensor data fusion^39^. Senthil proposed a PSO-based method to enhance the lifespan of wireless sensor networks^40^. Wang introduced a novel resampling PSO to improve sensor network performance^41^. Moreover, PSO can optimize cooperative working strategies, energy usage strategies, and sensor co-working strategies.

There are also a lot of researcher inspired from natural groups. Mirjalili citeMirjalili2014 proposed the gray wolf optimizer (GWO) in 2014. Heidari2019^42^ proposed the Harris hawks optimization (HHO) in 2021. Emary proposed the Abdollahzadeh proposed the african vultures optimization algorithm and artificial gorilla troops optimizer in 2021. Xue^43^ proposed theDung beetle optimizer (DBO) in 2023 As technology advances, applied research, such as sensor deployment and sensor data transmission, becomes increasingly high-dimensional and complex. To address this challenge, this paper delves into the characteristics of high-dimensional space and introduces a new nature-inspired metaheuristic algorithm: the Pair Barracuda Swarm Optimization Algorithm (PBSO).

Barracudas are highly social marine mammals that typically form large groups known as pods. The arrangement of sensors can draw inspiration from the distribution of these pods. The size of barracuda pods varies depending on the species and their environment. Common barracuda pods generally consist of a few dozen to a few hundred individuals, while king barracuda pods can number over a thousand individuals. These pods exhibit a strict social structure, typically led by a male, with females and juveniles comprising the rest. The leader of the barracuda pod guides the group’s movements, food search, and other activities. Communication within barracuda groups involves various methods, including sounds, body language, and physical contact. Barracudas emit high-frequency calls that can travel significant distances, aiding in underwater communication and navigation. Barracudas frequently cooperate in activities such as fishing, protecting their young, and defending against predators. They also form supersets to hunt large fish and cetaceans collectively. Barracudas display high intelligence and learning capabilities, enabling them to use tools for obtaining food, such as fishing with hooks, and collaborate with humans in tasks like rescue operations and marine research.

The main contributions of this paper are as follows:

- A novel evolution strategy is proposed in this paper to balance the global and local search ability of the algorithm. A Gaussian distribution is used in future position selection of particle units.
- Deep memory mechanism is introduced to enhance the global optimum escaping ability of the barracuda swarm.
- A barracuda pairs evolution model is designed to increase the optimization precision.

The rest of this paper is organized as follows: Section "Materials and methods" introduces details of the proposed method; Section "Results" introduces experiments and discussion; Section "Conclusions" presents the conclusion of this work.

## Materials and methods

### Barracudas swarms in nature

Survival resources within a barracuda population encompass necessities like food, water, and resting places. It is essential to distribute these resources effectively to cater to the needs of the entire population. In a barracuda swarm, the leader plays a pivotal role. The leader’s responsibilities include guiding the direction of the search and allocating resources. In some barracuda groups, the leader determines the group’s movement direction and coordinates activities such as food acquisition, thereby facilitating the collective access to survival resources. Typically, the leader barracuda enjoys priority access to food and resting places, while other group members adhere to group-defined allocation rules. In certain instances, barracudas engage in competitive resource allocation. This communication and resource allocation behavior in barracudas offers novel insights for sensor deployment strategies. For example, when searching for food, swifter or more skilled hunters among the barracudas may receive larger portions. In such cases, weaker members of the group may need to rely on cooperation and assistance to secure survival resources. Nonetheless, nature also illustrates instances of cooperative allocation and equitable distribution. In their exploration of barracuda population structures, researchers have observed that barracudas typically reside in pods. These life patterns can vary depending on the region, species, and season, but they often exhibit common patterns.

- barracuda groups: Most barracudas live in groups, usually consisting of dozens to hundreds of barracudas. Within these groups, barracudas often collaborate to feed, move and breed.
- barracuda Pairs: During the breeding season, some barracudas form pairs, consisting of a male and a female barracuda.
- Solitary barracudas: Some barracudas may also live alone, usually because they have been expelled or separated from the group. These solitary barracudas may find a new group or continue to live alone.

In short, barracudas usually live in groups, which can consist of a few to hundreds of barracudas. barracudas often have close social bonds with each other and exhibit a variety of life patterns, including groups, families, pairs and solitude.

### Barracudas swarm optimization algorithm

Inspired by the social structure and team behavior of barracudas, a novel barracudas swarm optimization algorithm (PBSO) is proposed in this work. The minimum evolutionary unit in PBSO is the barracuda pair, which contains separate DNA but shared memory.

#### Roles and behaviors

In PBSO, four different roles builds a stable relationship to explore the global best point. Details of different roles are listed below:

- barracuda pair: two barracudas in a pair, the evolutionary process involves the barracuda exchanging information with the leader barracuda and acquiring new candidate positions. Then each barracuda pair is given two new candidate positions, and the barracuda pair selects the optimal one from the two historical optimal positions and the two candidate positions. In PBSO, the barracuda pair is the standard evolutionary unit. During the evolutionary process, each barracuda participates in the computation.
- Best barracuda pair: The best two barracudas formed a best barracuda pair.
- Solitary barracuda: One of the best barracudas in history, always following the leader barracuda.
- Leader barracuda: The best barracuda pair and the solitary barracuda. During the evolutionary process, the leader barracuda is an aggregation of three barracuda individuals.

In each iteration, every barracuda will try to move toward to the barracuda leader. The candidate position of a barracuda is calculated by Eq. (1).

1 $$\begin{aligned} \begin{array}{l} \alpha = (individuals + leaders)/2\\ \beta = |individuals - leaders|\\ d\_candi = Gausi(\alpha ,\ \beta ) \end{array} \end{aligned}$$

where *individuals* stands the barracudae pair in the swarm, *leader* are the best barracuda in the swarm, $Gausi(\alpha , \beta )$ is the Gaussian distribution with a mean $\alpha$ and a standard deviation $\beta$.

#### Deep memory mechanism of the barracuda swarm optimization

To enhance the algorithm’s performance, we employ a deep memory mechanism that mimics the pairing behavior of barracudas. This mechanism involves two types of individuals:

- Ordinary individuals: They possess the current position and a single layer of depth memory.
- Leader individuals: They have the current position and a more extensive memory with two layers of depth.

This approach faithfully replicates the social structure of a barracuda school. You can find the detailed specifics of this strategy in Eq. (2).

2 $$\begin{aligned} \begin{array}{l} individuals=(memory_1, memory_2)\\ leaders=(leader_memory_1, leader_memory_2, leader_memory_3)\\ d\_candi = Gausi(\alpha ,\ \beta ) \end{array} \end{aligned}$$

where *individuals* stands for the normal barracudaes, the *leaders* stands for the leader of the barracuda swarm. Building upon this hierarchical structure, the algorithm simultaneously generates six candidate positions when calculating individual barracudae positions using Eq. (1). Subsequently, the algorithm identifies the top two positions from this pool for each barracudae individual. Once all barracuda individuals complete their updates, the algorithm can then determine the best two positions across the entire evolution. These two positions are combined with the best individuals from the previous round, resulting in five standout positions. Finally, the algorithm selects the top three positions to designate as the barracudae leader positions for this round.

#### The process, pseudo-code and flowchart of barracudas swarm optimization algorithm

**Figure 1:**
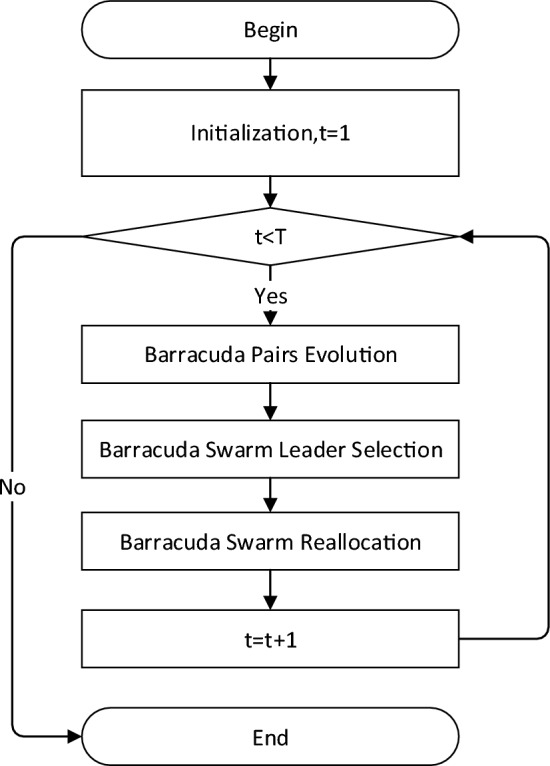
The flow chart of PBSO.

The PBSO includes three major process: barracuda pairs evolution, barracuda swarm leader selection and the barracuda swarm reallocation. The flowchart of PBSO is shown in Fig. 1. Details of all processes are summarized as follows:

- barracuda pairs evolution: Two barracudas entwined in pairs during the evolutionary process. They exchange information with the barracuda leader separately and rank themselves according to their fitness after updating their position.
- barracuda swarm leader selection: A support barracuda keeps following the leader barracuda pair. The support barracuda will engage into the evolutionary process, information exchanging, and position selection.
- barracuda swarm reallocation: In each iteration, each barracuda pair will generate six candidate positions with the barracuda swarm leader using Eq. (1). Then the top two positions will be selected as the new position of the barracuda pair. After all barracuda pairs get new positions, the barracuda swarm leader will update their positions with the new swam-best barracuda pair.
- barracuda leader: In each iteration, each barracuda pair will generate six candidate positions with the barracuda swarm leader using Eq. (1). Then the top two positions will be selected as the new position of the barracuda pair. After all barracuda pairs get new positions, the barracuda swarm leader will update their positions with the new swam-best barracuda pair.

## Results

### Experimental methods

To verify the optimization ability of proposed PBSO, the CEC2017 benchmark functions are used in validation test. The CEC2017 Benchmark Functions, also known as the IEEE Congress on Evolutionary Computation (CEC) 2017 Benchmark Functions, are a set of numerical optimization problems used to evaluate and benchmark the performance of optimization algorithms, particularly evolutionary algorithms. These benchmark functions were introduced as part of the CEC 2017 competition, which aimed to advance the field of optimization by providing a standardized set of challenging test problems. CEC2017 benchmark functions contain 4 types test functions:

- Unimodal Functions: fcuntion 1-2;
- Simple Multimodal Functions: fcuntion 3-9;
- Hybrid Functions: fcuntion 10-19;
- Composition Functions: fcuntion 20-29.

In order to validate the ability of PBSO to search in high-dimensional spaces, we used the highest dimensions of CEC2017. For the control group, we chose 2 classes of well-known algorithms. The first category is 5 state-of-the-art natural-inspired methods and the second category is 5 famous particle swarm-based algorithms.

### Comparison experiments with state-of-the-art natural-inspired methods

In this part, 5 state-of-the-art natural-inspired methods, including AVOA,DBO, GTO, GWO, and HHO, are tested with the CEC2017 benchmark functions. Experimental results are shown in Tables 1, 2, 3, 4 and 5. The mean, standard deviation, best and worst results of the 37 runs are recorded. Also, the Fridman test is implemented. The average rank and experimental parameters are shown in Table 5.

**Table 1:** Simulation results of AVOA, DBO, GTO, GWO, HHO and PBSO, $f_1$ to $f_{5}$.

Function	Data tpye	AVOA	DBO	GTO	GWO	HHO	PBSO
1	Mean	5.264E+03	6.846E+07	3.253E+09	3.051E+10	2.092E+08	1.361E+04
	STD	6.224E+03	3.725E+07	1.709E+09	6.571E+09	2.508E+07	2.234E+04
	Best	2.622E+00	7.918E+05	6.702E+08	1.511E+10	1.483E+08	1.552E−01
	Worst	3.062E+04	1.658E+08	6.911E+09	4.236E+10	2.589E+08	9.351E+04
	Rank	1	3	5	6	4	2
2	Mean	1.835E+31	2.131E+139	1.180E+127	1.840E+121	3.903E+73	1.154E+97
	STD	1.088E+32	1.296E+140	7.180E+127	8.396E+121	2.374E+74	7.021E+97
	Best	2.622E+00	7.918E+05	6.702E+08	1.511E+10	1.483E+08	1.552E−01
	Worst	3.062E+04	1.658E+08	6.911E+09	4.236E+10	2.589E+08	9.351E+04
	Rank	1	6	5	4	2	3
3	Mean	1.859E+04	3.140E+05	1.165E+06	1.995E+05	3.299E+04	6.820E+05
	STD	6.292E+03	2.447E+04	2.697E+06	2.143E+04	7.427E+03	5.250E+05
	Best	2.622E+00	7.918E+05	6.702E+08	1.511E+10	1.483E+08	1.552E−01
	Worst	3.062E+04	1.658E+08	6.911E+09	4.236E+10	2.589E+08	9.351E+04
	Rank	1	4	6	3	2	5
4	Mean	2.485E+02	4.353E+02	1.414E+03	2.252E+03	4.665E+02	1.462E+02
	STD	4.582E+01	9.396E+01	2.727E+02	7.312E+02	7.282E+01	3.496E+01
	Best	2.622E+00	7.918E+05	6.702E+08	1.511E+10	1.483E+08	1.552E−01
	Worst	3.062E+04	1.658E+08	6.911E+09	4.236E+10	2.589E+08	9.351E+04
	Rank	2	3	5	6	4	1
5	Mean	7.886E+02	1.091E+03	8.020E+02	5.405E+02	9.202E+02	6.785E+02
	STD	7.296E+01	1.581E+02	1.032E+02	6.500E+01	5.499E+01	1.279E+02
	Best	2.622E+00	7.918E+05	6.702E+08	1.511E+10	1.483E+08	1.552E−01
	Worst	3.062E+04	1.658E+08	6.911E+09	4.236E+10	2.589E+08	9.351E+04
	Rank	3	6	4	1	5	2

**Table 2:** Simulation results of AVOA, DBO, GTO, GWO, HHO and PBSO, $f_6$ to $f_{12}$.

Function	Data tpye	AVOA	DBO	GTO	GWO	HHO	PBSO
6	Mean	4.170E+01	6.806E+01	7.445E+01	2.586E+01	7.577E+01	3.839E+01
	STD	4.373E+00	9.030E+00	1.479E+01	4.981E+00	3.419E+00	8.915E+00
	Best	2.622E+00	7.918E+05	6.702E+08	1.511E+10	1.483E+08	1.552E−01
	Worst	3.062E+04	1.658E+08	6.911E+09	4.236E+10	2.589E+08	9.351E+04
	Rank	3	4	5	1	6	2
7	Mean	2.123E+03	1.521E+03	1.745E+03	1.022E+03	2.730E+03	7.912E+02
	STD	1.690E+02	3.473E+02	2.712E+02	1.131E+02	1.220E+02	1.367E+02
	Best	2.622E+00	7.918E+05	6.702E+08	1.511E+10	1.483E+08	1.552E−01
	Worst	3.062E+04	1.658E+08	6.911E+09	4.236E+10	2.589E+08	9.351E+04
	Rank	5	3	4	2	6	1
8	Mean	8.978E+02	1.158E+03	8.368E+02	5.404E+02	1.037E+03	7.330E+02
	STD	8.606E+01	1.868E+02	1.680E+02	6.259E+01	7.166E+01	1.527E+02
	Best	2.622E+00	7.918E+05	6.702E+08	1.511E+10	1.483E+08	1.552E−01
	Worst	3.062E+04	1.658E+08	6.911E+09	4.236E+10	2.589E+08	9.351E+04
	Rank	4	6	3	1	5	2
9	Mean	2.177E+04	3.655E+04	5.218E+04	2.088E+04	2.932E+04	2.887E+04
	STD	1.385E+03	1.019E+04	2.116E+04	9.788E+03	3.416E+03	9.101E+03
	Best	2.622E+00	7.918E+05	6.702E+08	1.511E+10	1.483E+08	1.552E−01
	Worst	3.062E+04	1.658E+08	6.911E+09	4.236E+10	2.589E+08	9.351E+04
	Rank	2	5	6	1	4	3
10	Mean	1.523E+04	1.711E+04	2.656E+04	1.339E+04	1.745E+04	1.641E+04
	STD	1.747E+03	2.183E+03	6.264E+03	1.336E+03	1.580E+03	5.769E+03
	Best	2.622E+00	7.918E+05	6.702E+08	1.511E+10	1.483E+08	1.552E−01
	Worst	3.062E+04	1.658E+08	6.911E+09	4.236E+10	2.589E+08	9.351E+04
	Rank	2	4	6	1	5	3
11	Mean	1.241E+03	1.100E+04	9.393E+04	3.726E+04	1.923E+03	4.984E+02
	STD	2.125E+02	1.175E+04	1.453E+05	9.118E+03	2.395E+02	1.800E+02
	Best	2.622E+00	7.918E+05	6.702E+08	1.511E+10	1.483E+08	1.552E−01
	Worst	3.062E+04	1.658E+08	6.911E+09	4.236E+10	2.589E+08	9.351E+04
	Rank	2	4	6	5	3	1
12	Mean	1.129E+07	4.880E+08	3.921E+08	4.746E+09	2.729E+08	9.332E+06
	STD	4.803E+06	2.824E+08	1.840E+08	2.502E+09	6.969E+07	3.408E+06
	Best	2.622E+00	7.918E+05	6.702E+08	1.511E+10	1.483E+08	1.552E−01
	Worst	3.062E+04	1.658E+08	6.911E+09	4.236E+10	2.589E+08	9.351E+04
	Rank	2	5	4	6	3	1

**Table 3:** Simulation results of AVOA, DBO, GTO, GWO, HHO and PBSO, $f_{13}$ to $f_{19}$.

Function	Data tpye	AVOA	DBO	GTO	GWO	HHO	PBSO
13	Mean	4.516E+04	6.794E+06	2.840E+04	3.244E+08	3.305E+06	9.243E+03
	STD	1.080E+04	8.399E+06	1.145E+04	2.932E+08	1.037E+06	1.123E+04
	Best	2.622E+00	7.918E+05	6.702E+08	1.511E+10	1.483E+08	1.552E−01
	Worst	3.062E+04	1.658E+08	6.911E+09	4.236E+10	2.589E+08	9.351E+04
	Rank	3	5	2	6	4	1
14	Mean	1.967E+05	5.406E+06	7.537E+05	3.318E+06	8.902E+05	2.475E+05
	STD	8.369E+04	5.249E+06	4.854E+05	1.865E+06	2.941E+05	1.202E+05
	Best	2.622E+00	7.918E+05	6.702E+08	1.511E+10	1.483E+08	1.552E−01
	Worst	3.062E+04	1.658E+08	6.911E+09	4.236E+10	2.589E+08	9.351E+04
	Rank	1	6	3	5	4	2
15	Mean	2.298E+04	1.255E+06	6.464E+03	3.429E+07	9.133E+05	8.271E+03
	STD	9.763E+03	2.150E+06	3.552E+03	5.476E+07	3.037E+05	1.242E+04
	Best	2.622E+00	7.918E+05	6.702E+08	1.511E+10	1.483E+08	1.552E−01
	Worst	3.062E+04	1.658E+08	6.911E+09	4.236E+10	2.589E+08	9.351E+04
	Rank	3	5	1	6	4	2
16	Mean	4.741E+03	6.090E+03	5.968E+03	3.968E+03	5.462E+03	4.686E+03
	STD	7.264E+02	1.021E+03	1.974E+03	5.655E+02	6.696E+02	9.264E+02
	Best	2.622E+00	7.918E+05	6.702E+08	1.511E+10	1.483E+08	1.552E−01
	Worst	3.062E+04	1.658E+08	6.911E+09	4.236E+10	2.589E+08	9.351E+04
	Rank	3	6	5	1	4	2
17	Mean	4.152E+03	5.112E+03	3.722E+03	2.778E+03	4.439E+03	4.051E+03
	STD	7.030E+02	8.359E+02	7.296E+02	4.274E+02	7.151E+02	6.889E+02
	Best	2.622E+00	7.918E+05	6.702E+08	1.511E+10	1.483E+08	1.552E−01
	Worst	3.062E+04	1.658E+08	6.911E+09	4.236E+10	2.589E+08	9.351E+04
	Rank	4	6	2	1	5	3
18	Mean	3.622E+05	6.755E+06	1.559E+06	3.610E+06	1.940E+06	1.331E+06
	STD	1.312E+05	4.544E+06	8.807E+05	4.031E+06	7.811E+05	7.441E+05
	Best	2.622E+00	7.918E+05	6.702E+08	1.511E+10	1.483E+08	1.552E−01
	Worst	3.062E+04	1.658E+08	6.911E+09	4.236E+10	2.589E+08	9.351E+04
	Rank	1	6	3	5	4	2
19	Mean	1.120E+04	1.784E+06	1.539E+04	8.358E+07	3.840E+06	1.575E+04
	STD	8.368E+03	1.703E+06	1.481E+04	8.375E+07	1.451E+06	1.898E+04
	Best	2.622E+00	7.918E+05	6.702E+08	1.511E+10	1.483E+08	1.552E−01
	Worst	3.062E+04	1.658E+08	6.911E+09	4.236E+10	2.589E+08	9.351E+04
	Rank	1	4	2	6	5	3

**Table 4:** Simulation results of AVOA, DBO, GTO, GWO, HHO and PBSO, $f_{20}$ to $f_{25}$.

Function	Data tpye	AVOA	DBO	GTO	GWO	HHO	PBSO
20	Mean	3.700E+03	3.910E+03	4.468E+03	2.479E+03	3.651E+03	2.938E+03
	STD	6.451E+02	5.399E+02	1.196E+03	8.164E+02	4.507E+02	5.002E+02
	Best	2.622E+00	7.918E+05	6.702E+08	1.511E+10	1.483E+08	1.552E−01
	Worst	3.062E+04	1.658E+08	6.911E+09	4.236E+10	2.589E+08	9.351E+04
	Rank	4	5	6	1	3	2
21	Mean	1.325E+03	1.471E+03	9.730E+02	7.552E+02	1.692E+03	9.391E+02
	STD	1.634E+02	1.120E+02	1.926E+02	6.552E+01	1.921E+02	1.214E+02
	Best	2.622E+00	7.918E+05	6.702E+08	1.511E+10	1.483E+08	1.552E−01
	Worst	3.062E+04	1.658E+08	6.911E+09	4.236E+10	2.589E+08	9.351E+04
	Rank	4	5	3	1	6	2
22	Mean	1.713E+04	1.809E+04	2.724E+04	1.503E+04	2.005E+04	1.832E+04
	STD	1.148E+03	2.329E+03	7.070E+03	1.538E+03	1.516E+03	6.461E+03
	Best	2.622E+00	7.918E+05	6.702E+08	1.511E+10	1.483E+08	1.552E−01
	Worst	3.062E+04	1.658E+08	6.911E+09	4.236E+10	2.589E+08	9.351E+04
	Rank	2	3	6	1	5	4
23	Mean	1.453E+03	1.794E+03	1.494E+03	1.133E+03	2.207E+03	1.218E+03
	STD	1.544E+02	1.931E+02	2.519E+02	6.302E+01	1.452E+02	9.126E+01
	Best	2.622E+00	7.918E+05	6.702E+08	1.511E+10	1.483E+08	1.552E−01
	Worst	3.062E+04	1.658E+08	6.911E+09	4.236E+10	2.589E+08	9.351E+04
	Rank	3	5	4	1	6	2
24	Mean	2.343E+03	2.614E+03	1.996E+03	1.513E+03	3.209E+03	1.730E+03
	STD	2.019E+02	2.967E+02	2.445E+02	9.075E+01	3.176E+02	1.642E+02
	Best	2.622E+00	7.918E+05	6.702E+08	1.511E+10	1.483E+08	1.552E−01
	Worst	3.062E+04	1.658E+08	6.911E+09	4.236E+10	2.589E+08	9.351E+04
	Rank	4	5	3	1	6	2
25	Mean	7.947E+02	1.779E+03	1.775E+03	2.858E+03	1.027E+03	7.646E+02
	STD	6.671E+01	2.724E+03	2.549E+02	5.274E+02	7.607E+01	6.212E+01
	Best	2.622E+00	7.918E+05	6.702E+08	1.511E+10	1.483E+08	1.552E−01
	Worst	3.062E+04	1.658E+08	6.911E+09	4.236E+10	2.589E+08	9.351E+04
	Rank	2	5	4	6	3	1

**Table 5:** Simulation results of AVOA, DBO, GTO, GWO, HHO and PBSO, $f_{26}$ to $f_{29}$.

Function	Data tpye	AVOA	DBO	GTO	GWO	HHO	PBSO
26	Mean	1.804E+04	1.823E+04	1.559E+04	9.732E+03	2.163E+04	1.283E+04
	STD	2.387E+03	3.383E+03	3.643E+03	7.191E+02	1.612E+03	1.541E+03
	Best	2.622E+00	7.918E+05	6.702E+08	1.511E+10	1.483E+08	1.552E−01
	Worst	3.062E+04	1.658E+08	6.911E+09	4.236E+10	2.589E+08	9.351E+04
	Rank	4	5	3	1	6	2
27	Mean	1.266E+03	1.251E+03	1.096E+03	1.146E+03	1.440E+03	5.000E+02
	STD	2.335E+02	1.965E+02	1.521E+02	1.168E+02	2.943E+02	3.608E−04
	Best	2.622E+00	7.918E+05	6.702E+08	1.511E+10	1.483E+08	1.552E−01
	Worst	3.062E+04	1.658E+08	6.911E+09	4.236E+10	2.589E+08	9.351E+04
	Rank	5	4	2	3	6	1
28	Mean	5.586E+02	1.294E+04	1.900E+03	4.007E+03	7.823E+02	5.000E+02
	STD	3.210E+01	7.025E+03	4.383E+02	1.053E+03	4.301E+01	6.068E−04
	Best	2.622E+00	7.918E+05	6.702E+08	1.511E+10	1.483E+08	1.552E−01
	Worst	3.062E+04	1.658E+08	6.911E+09	4.236E+10	2.589E+08	9.351E+04
	Rank	2	6	4	5	3	1
29	Mean	4.859E+03	6.001E+03	6.526E+03	4.320E+03	5.855E+03	4.005E+03
	STD	6.071E+02	1.183E+03	1.401E+03	4.799E+02	6.816E+02	7.758E+02
	Best	2.622E+00	7.918E+05	6.702E+08	1.511E+10	1.483E+08	1.552E−01
	Worst	3.062E+04	1.658E+08	6.911E+09	4.236E+10	2.589E+08	9.351E+04
	Rank	3	5	6	2	4	1
Average Rank		2.6552	4.7931	4.069	3.069	4.3793	2.0345
Population Size: 100							
Max Iteration times: 10000							
Dimension: 100							
Search Range: [−100 100]							
Independent Runs: 37							

**Figure 2:**
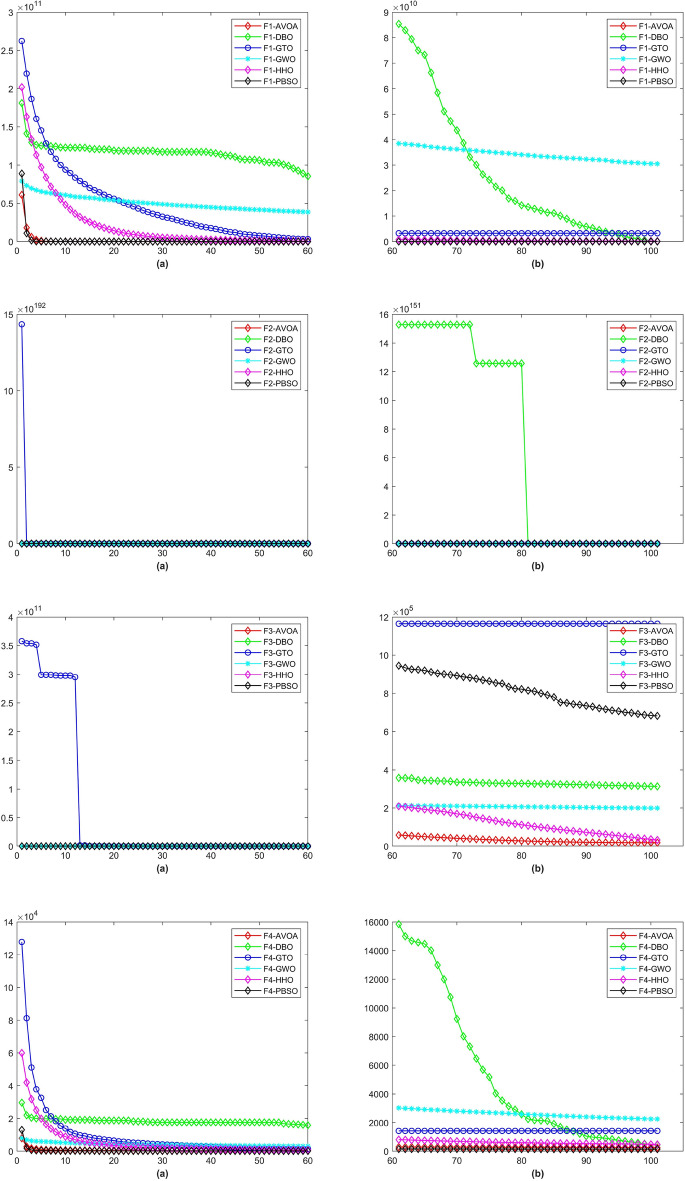
The CD curve of $f_{1-4}$ for BBPSO, DLSBBPSO, PBBPSO, TBBPSO, ETBBPSO and PBSO.

**Figure 3:**
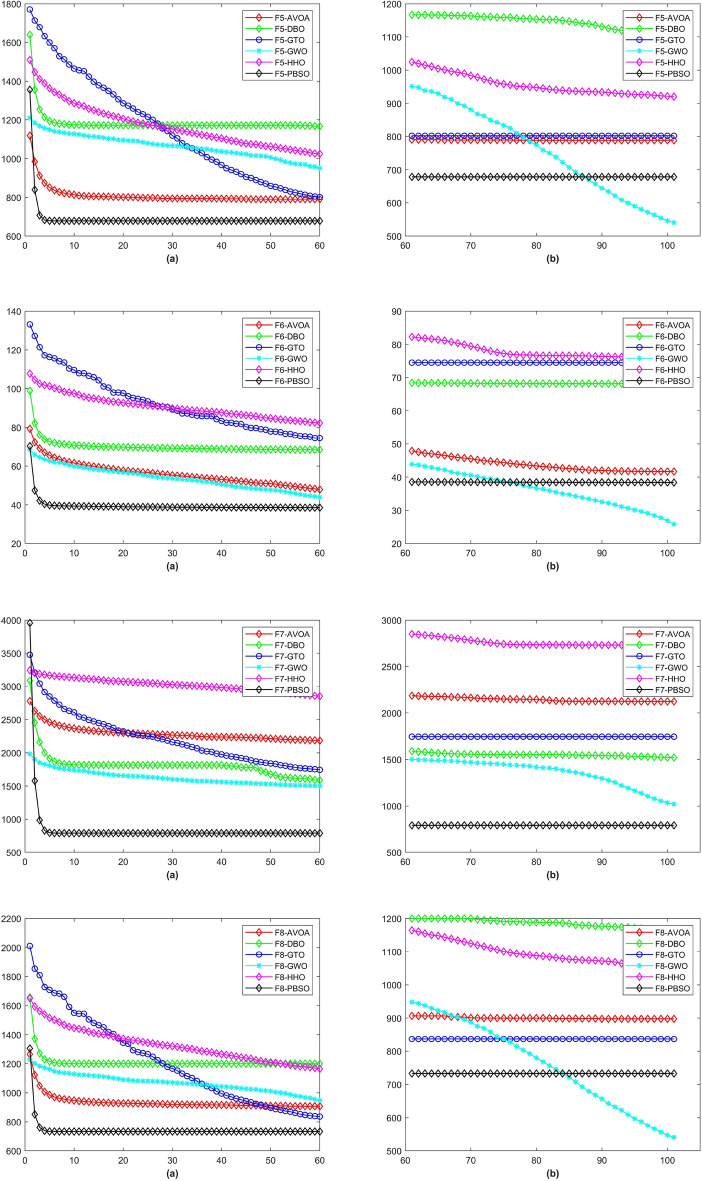
The CD curve of $f_{5-8}$ for BBPSO, DLSBBPSO, PBBPSO, TBBPSO, ETBBPSO and PBSO.

**Figure 4:**
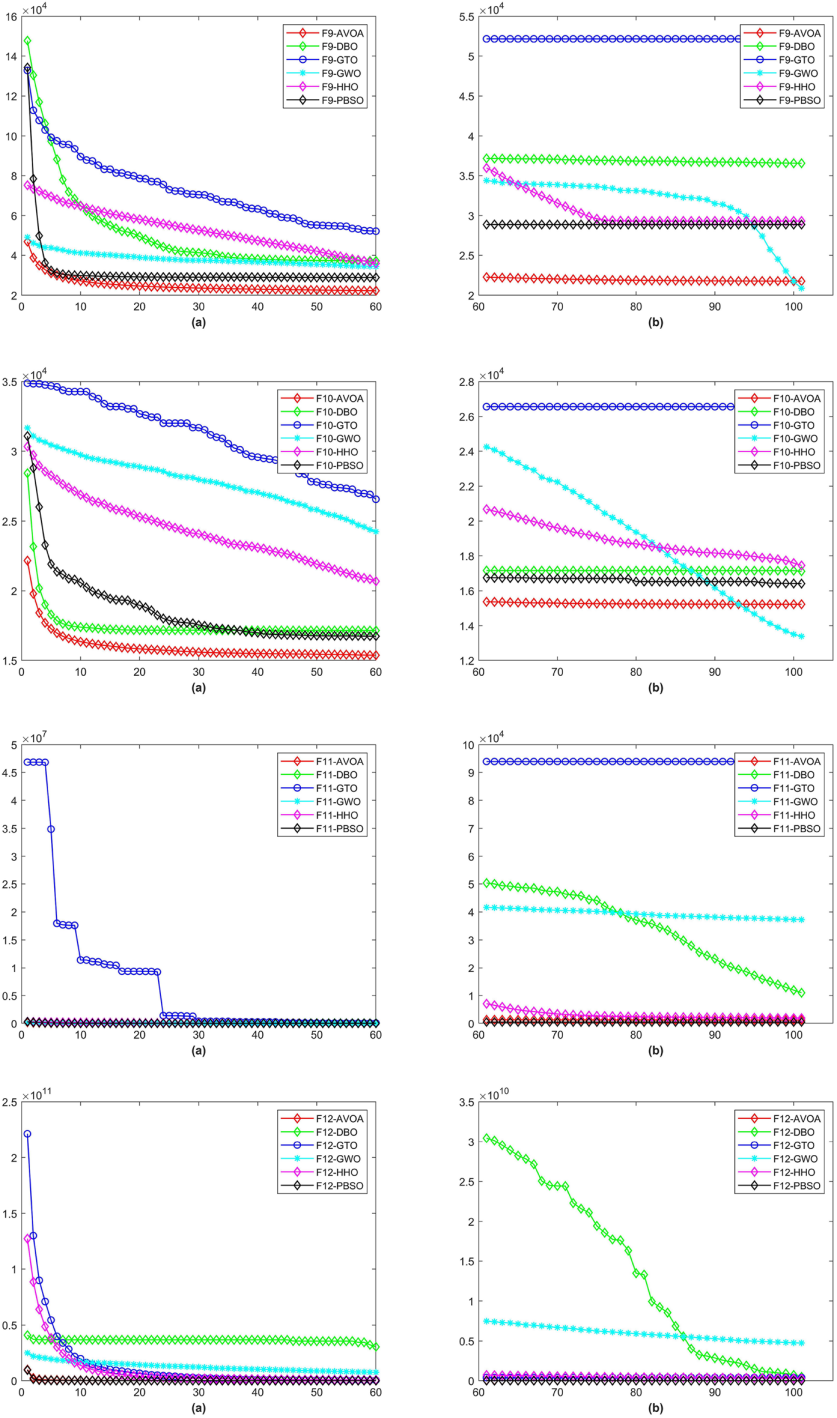
The CD curve of $f_{9-12}$ for BBPSO, DLSBBPSO, PBBPSO, TBBPSO, ETBBPSO and PBSO.

**Figure 5:**
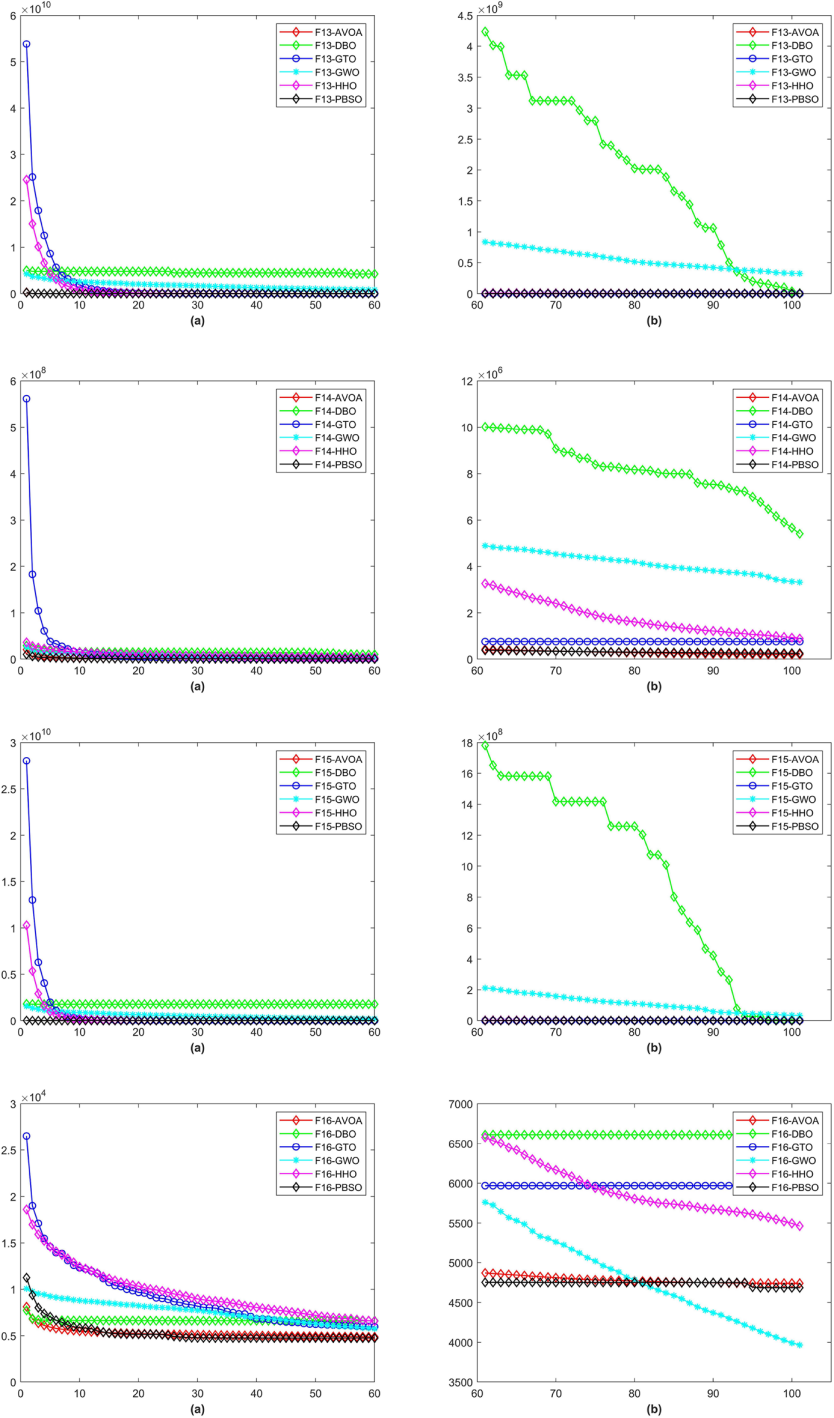
The CD curve of $f_{13-16}$ for BBPSO, DLSBBPSO, PBBPSO, TBBPSO, ETBBPSO and PBSO.

**Figure 6:**
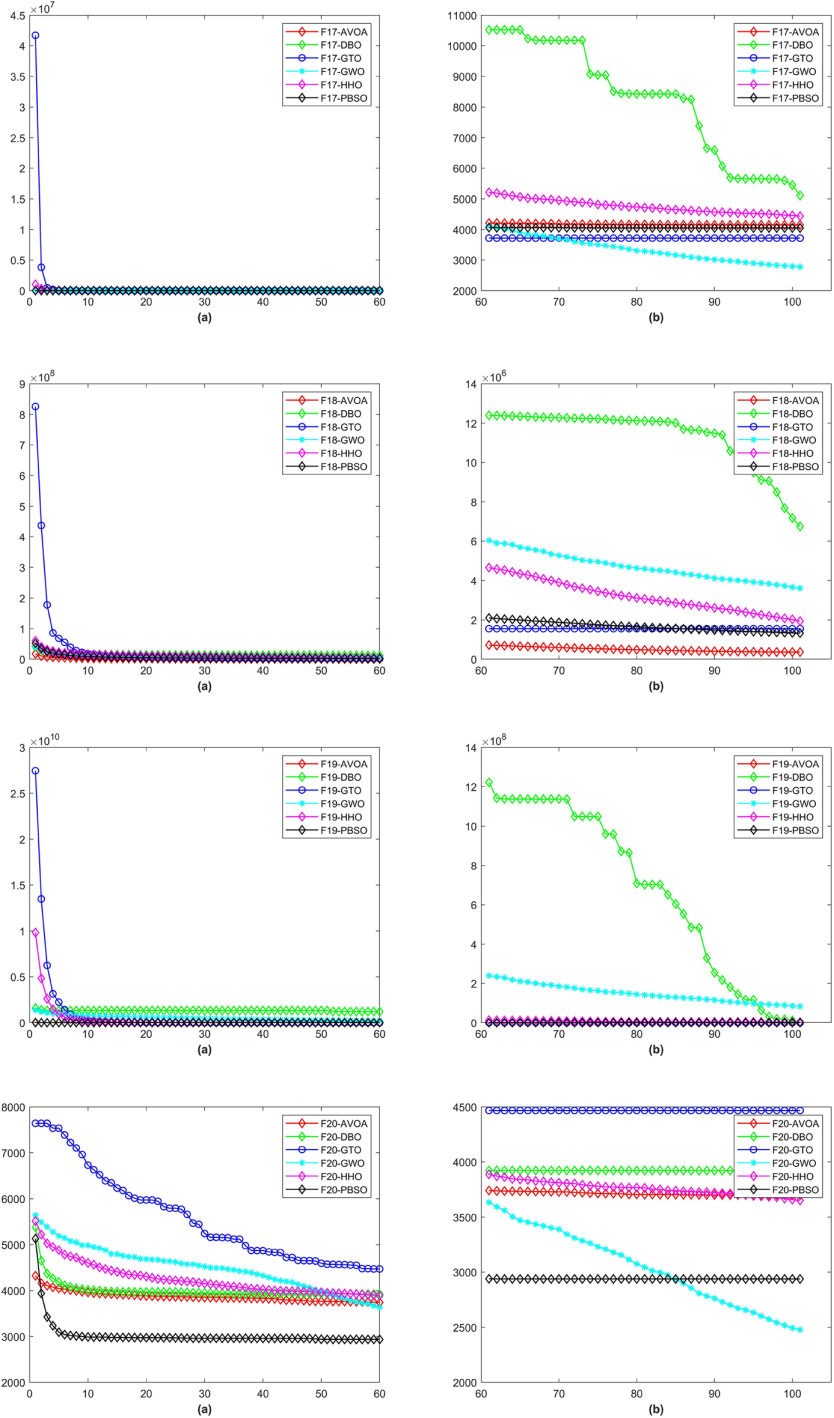
The CD curve of $f_{17-20}$ for BBPSO, DLSBBPSO, PBBPSO, TBBPSO, ETBBPSO and PBSO.

**Figure 7:**
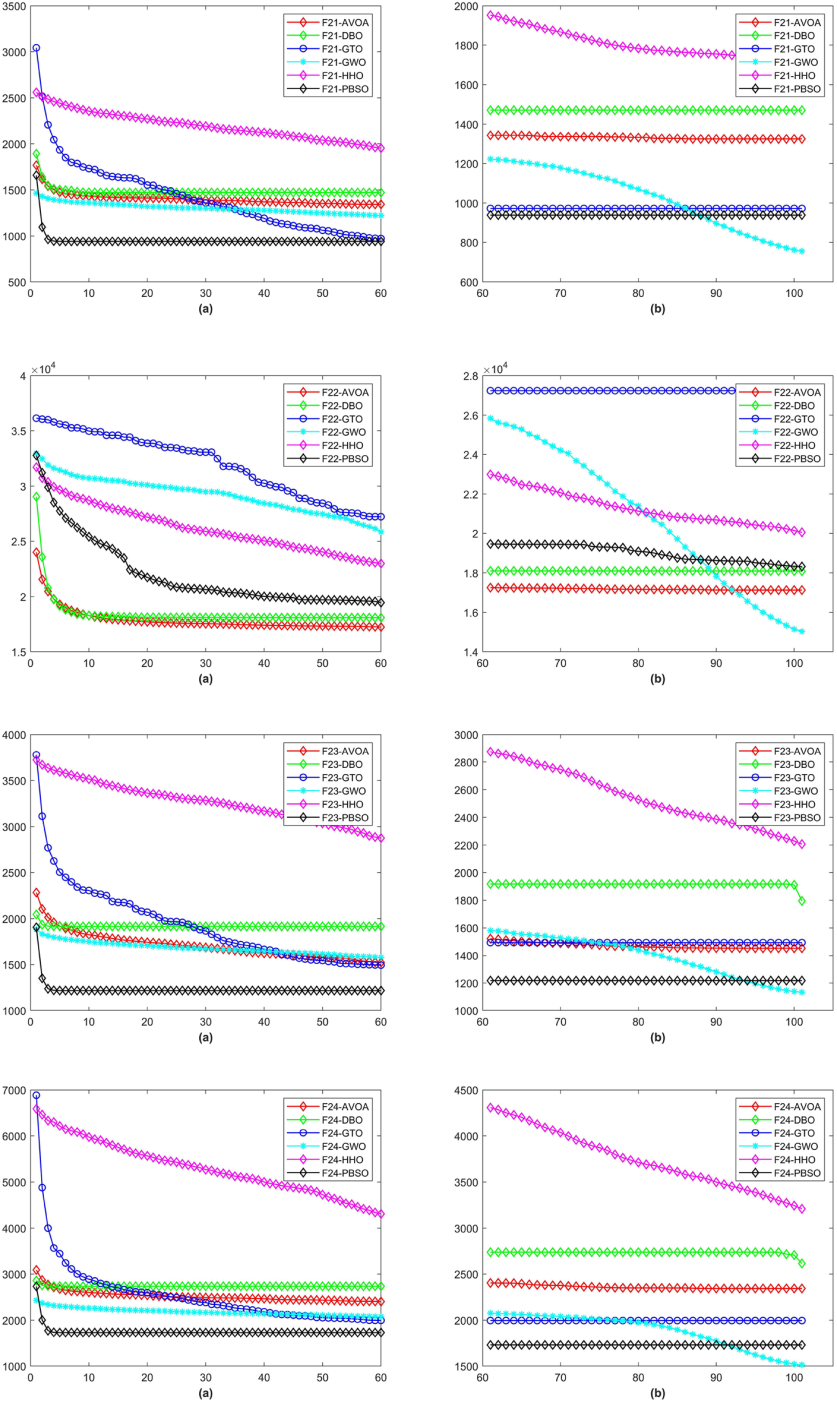
The CD curve of $f_{21-24}$ for BBPSO, DLSBBPSO, PBBPSO, TBBPSO, ETBBPSO and PBSO.

**Figure 8:**
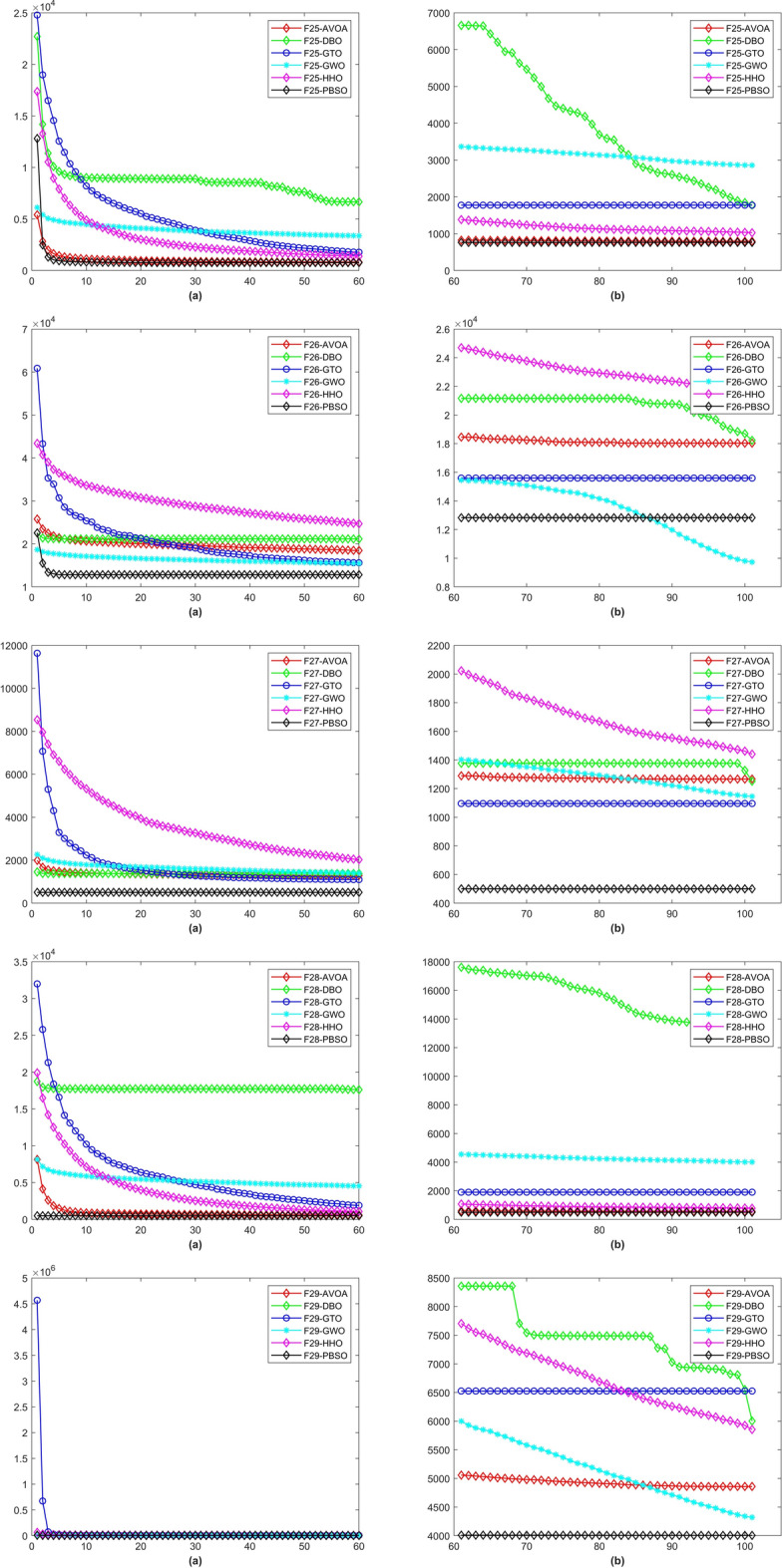
The CD curve of $f_{25-29}$ for BBPSO, DLSBBPSO, PBBPSO, TBBPSO, ETBBPSO and PBSO.

In a total of 29 test functions, PBSO have 9 firsts, 13 seconds, 5 thirds, 1 fourth and 1 fifth. The average rank is 2.0345. PBSO performs the best among all tested algorithms and is able to consistently provide high-precision solutions to high-dimensional optimization problems. Compared withe other state-of-the-are natural-inspired methods, PBSO does not require pre-training of parameters and does not have complex control functions. The structure of the population and the adaptive evolutionary strategy provide an excellent local optimal escape ability for PBSO. Furthermore, the local organization of the PBSO algorithm enables the population to explore information more efficiently in high-dimensional spaces. At the same time, the deep memory mechanism equips the barracuda swarm with a stronger ability to escape local optima. To demonstrate the convergence capability of test algorithms in more detail, convergence diagram (CD) are shown in Figs. 2, 3, 4, 5, 6, 7 and 8.

### Comparison experiments with PSO-based methods

In this part, the standard BBPSO, DLSBBPSO, PBBPSO, TBBPSO, and ETBBPSO are used in control group. The mean, standard deviation, best and worst results of the 37 runs are recorded in Tables 6, 7, 8, 9, and 10. In a total of 29 test functions, PBSO gets 23 firsts, 2 seconds, 1 thirds, 2 fourths, and 1 sixths, the average rank is 1.52. Also, the Fridman test is implemented. The average rank and experimental parameters are shown in Table 10.

**Table 6:** Simulation results of BBPSO, DLSBBPSO, ETBBPSO, PBBPSO, TBBPSO and PBSO, $f_1$ to $f_{6}$.

Function	Data type	BBPSO	DLSBBPSO	ETBBPSO	PBBPSO	TBBPSO	PBSO
1	Mean	1.373E+04	1.240E+04	3.920E+04	1.153E+04	3.420E+04	1.361E+04
	STD	1.596E+04	1.518E+04	2.941E+04	1.644E+04	3.368E+04	2.234E+04
	Best	6.060E-02	2.048E+00	6.230E+02	2.932E+01	2.250E+02	1.552E−01
	Worst	5.915E+04	5.297E+04	1.256E+05	5.966E+04	1.359E+05	9.351E+04
	Rank	4	2	6	1	5	3
2	Mean	6.847E+120	2.275E+123	8.409E+121	1.060E+136	1.196E+127	1.154E+97
	STD	4.165E+121	9.873E+123	5.115E+122	6.450E+136	7.277E+127	7.021E+97
	Best	6.060E−02	2.048E+00	6.230E+02	2.932E+01	2.250E+02	1.552E−01
	Worst	5.915E+04	5.297E+04	1.256E+05	5.966E+04	1.359E+05	9.351E+04
	Rank	2	4	3	6	5	1
3	Mean	3.313E+06	3.755E+06	2.583E+06	3.356E+06	1.852E+06	6.820E+05
	STD	3.245E+06	2.255E+06	1.911E+06	2.802E+06	9.388E+05	5.250E+05
	Best	6.060E−02	2.048E+00	6.230E+02	2.932E+01	2.250E+02	1.552E−01
	Worst	5.915E+04	5.297E+04	1.256E+05	5.966E+04	1.359E+05	9.351E+04
	Rank	4	6	3	5	2	1
4	Mean	1.677E+02	1.628E+02	1.680E+02	1.603E+02	1.697E+02	1.462E+02
	STD	6.518E+01	4.412E+01	5.554E+01	4.725E+01	5.709E+01	3.496E+01
	Best	6.060E−02	2.048E+00	6.230E+02	2.932E+01	2.250E+02	1.552E−01
	Worst	5.915E+04	5.297E+04	1.256E+05	5.966E+04	1.359E+05	9.351E+04
	Rank	4	3	5	2	6	1
5	Mean	9.114E+02	8.541E+02	9.123E+02	9.028E+02	9.573E+02	6.785E+02
	STD	1.393E+02	1.704E+02	1.773E+02	1.660E+02	1.589E+02	1.279E+02
	Best	6.060E−02	2.048E+00	6.230E+02	2.932E+01	2.250E+02	1.552E−01
	Worst	5.915E+04	5.297E+04	1.256E+05	5.966E+04	1.359E+05	9.351E+04
	Rank	4	2	5	3	6	1
6	Mean	4.099E+01	4.166E+01	4.143E+01	3.981E+01	3.569E+01	3.839E+01
	STD	8.319E+00	8.126E+00	9.194E+00	7.800E+00	6.829E+00	8.915E+00
	Best	6.060E−02	2.048E+00	6.230E+02	2.932E+01	2.250E+02	1.552E−01
	Worst	5.915E+04	5.297E+04	1.256E+05	5.966E+04	1.359E+05	9.351E+04
	Rank	4	6	5	3	1	2

**Table 7:** Simulation results of BBPSO, DLSBBPSO, ETBBPSO, PBBPSO, TBBPSO and PBSO, $f_7$ to $f_{13}$.

Function	Data type	BBPSO	DLSBBPSO	ETBBPSO	PBBPSO	TBBPSO	PBSO
7	Mean	9.389E+02	8.513E+02	8.577E+02	8.988E+02	9.100E+02	7.912E+02
	STD	1.910E+02	1.493E+02	1.266E+02	1.514E+02	1.313E+02	1.367E+02
	Best	6.060E−02	2.048E+00	6.230E+02	2.932E+01	2.250E+02	1.552E−01
	Worst	5.915E+04	5.297E+04	1.256E+05	5.966E+04	1.359E+05	9.351E+04
	Rank	6	2	3	4	5	1
8	Mean	8.538E+02	7.789E+02	9.109E+02	9.793E+02	9.552E+02	7.330E+02
	STD	1.678E+02	1.646E+02	1.674E+02	2.023E+02	1.655E+02	1.527E+02
	Best	6.060E−02	2.048E+00	6.230E+02	2.932E+01	2.250E+02	1.552E−01
	Worst	5.915E+04	5.297E+04	1.256E+05	5.966E+04	1.359E+05	9.351E+04
	Rank	3	2	4	6	5	1
9	Mean	3.614E+04	3.030E+04	3.635E+04	4.016E+04	3.904E+04	2.887E+04
	STD	6.494E+03	1.476E+04	1.451E+04	1.223E+04	1.155E+04	9.101E+03
	Best	6.060E−02	2.048E+00	6.230E+02	2.932E+01	2.250E+02	1.552E−01
	Worst	5.915E+04	5.297E+04	1.256E+05	5.966E+04	1.359E+05	9.351E+04
	Rank	3	2	4	6	5	1
10	Mean	2.349E+04	3.019E+04	2.166E+04	3.136E+04	2.467E+04	1.641E+04
	STD	9.064E+03	5.276E+03	8.357E+03	4.805E+03	5.289E+03	5.769E+03
	Best	6.060E−02	2.048E+00	6.230E+02	2.932E+01	2.250E+02	1.552E−01
	Worst	5.915E+04	5.297E+04	1.256E+05	5.966E+04	1.359E+05	9.351E+04
	Rank	3	5	2	6	4	1
11	Mean	1.622E+03	6.155E+03	4.106E+03	6.668E+03	4.737E+03	4.984E+02
	STD	3.266E+03	7.188E+03	3.390E+03	7.626E+03	5.101E+03	1.800E+02
	Best	6.060E−02	2.048E+00	6.230E+02	2.932E+01	2.250E+02	1.552E−01
	Worst	5.915E+04	5.297E+04	1.256E+05	5.966E+04	1.359E+05	9.351E+04
	Rank	2	5	3	6	4	1
12	Mean	5.514E+07	6.026E+07	6.148E+07	6.217E+07	4.700E+07	9.332E+06
	STD	2.463E+07	3.261E+07	2.723E+07	2.755E+07	2.981E+07	3.408E+06
	Best	6.060E−02	2.048E+00	6.230E+02	2.932E+01	2.250E+02	1.552E−01
	Worst	5.915E+04	5.297E+04	1.256E+05	5.966E+04	1.359E+05	9.351E+04
	Rank	3	4	5	6	2	1
13	Mean	1.119E+04	1.124E+04	1.062E+04	7.701E+03	1.501E+04	9.243E+03
	STD	1.324E+04	1.351E+04	1.586E+04	9.208E+03	1.881E+04	1.123E+04
	Best	6.060E−02	2.048E+00	6.230E+02	2.932E+01	2.250E+02	1.552E−01
	Worst	5.915E+04	5.297E+04	1.256E+05	5.966E+04	1.359E+05	9.351E+04
	Rank	4	5	3	1	6	2

**Table 8:** Simulation results of BBPSO, DLSBBPSO, ETBBPSO, PBBPSO, TBBPSO and PBSO, $f_{14}$ to $f_{20}$.

Function	Data type	BBPSO	DLSBBPSO	ETBBPSO	PBBPSO	TBBPSO	PBSO
14	Mean	1.180E+06	1.117E+06	1.199E+06	1.236E+06	1.111E+06	2.475E+05
	STD	7.376E+05	5.938E+05	6.039E+05	8.928E+05	6.803E+05	1.202E+05
	Best	6.060E−02	2.048E+00	6.230E+02	2.932E+01	2.250E+02	1.552E−01
	Worst	5.915E+04	5.297E+04	1.256E+05	5.966E+04	1.359E+05	9.351E+04
	Rank	4	3	5	6	2	1
15	Mean	1.002E+04	5.199E+03	4.683E+03	6.903E+03	8.981E+03	8.271E+03
	STD	1.381E+04	6.831E+03	6.419E+03	6.515E+03	1.140E+04	1.242E+04
	Best	6.060E−02	2.048E+00	6.230E+02	2.932E+01	2.250E+02	1.552E−01
	Worst	5.915E+04	5.297E+04	1.256E+05	5.966E+04	1.359E+05	9.351E+04
	Rank	6	2	1	3	5	4
16	Mean	5.538E+03	9.788E+03	6.447E+03	9.839E+03	7.192E+03	4.686E+03
	STD	1.689E+03	2.347E+03	2.338E+03	2.297E+03	2.475E+03	9.264E+02
	Best	6.060E−02	2.048E+00	6.230E+02	2.932E+01	2.250E+02	1.552E−01
	Worst	5.915E+04	5.297E+04	1.256E+05	5.966E+04	1.359E+05	9.351E+04
	Rank	2	5	3	6	4	1
17	Mean	4.682E+03	5.703E+03	4.735E+03	6.240E+03	4.964E+03	4.051E+03
	STD	8.287E+02	1.625E+03	1.096E+03	1.598E+03	1.060E+03	6.889E+02
	Best	6.060E−02	2.048E+00	6.230E+02	2.932E+01	2.250E+02	1.552E−01
	Worst	5.915E+04	5.297E+04	1.256E+05	5.966E+04	1.359E+05	9.351E+04
	Rank	2	5	3	6	4	1
18	Mean	5.431E+06	7.872E+06	6.770E+06	6.622E+06	5.146E+06	1.331E+06
	STD	4.000E+06	5.038E+06	4.099E+06	4.992E+06	2.571E+06	7.441E+05
	Best	6.060E−02	2.048E+00	6.230E+02	2.932E+01	2.250E+02	1.552E−01
	Worst	5.915E+04	5.297E+04	1.256E+05	5.966E+04	1.359E+05	9.351E+04
	Rank	3	6	5	4	2	1
19	Mean	1.312E+04	7.559E+03	1.076E+04	9.473E+03	8.860E+03	1.575E+04
	STD	1.757E+04	1.150E+04	1.516E+04	1.190E+04	9.107E+03	1.898E+04
	Best	6.060E−02	2.048E+00	6.230E+02	2.932E+01	2.250E+02	1.552E−01
	Worst	5.915E+04	5.297E+04	1.256E+05	5.966E+04	1.359E+05	9.351E+04
	Rank	5	1	4	3	2	6
20	Mean	3.387E+03	4.615E+03	3.894E+03	5.091E+03	3.779E+03	2.938E+03
	STD	7.836E+02	1.434E+03	1.388E+03	1.312E+03	1.114E+03	5.002E+02
	Best	6.060E−02	2.048E+00	6.230E+02	2.932E+01	2.250E+02	1.552E−01
	Worst	5.915E+04	5.297E+04	1.256E+05	5.966E+04	1.359E+05	9.351E+04
	Rank	2	5	4	6	3	1

**Table 9:** Simulation results of BBPSO, DLSBBPSO, ETBBPSO, PBBPSO, TBBPSO and PBSO, $f_{21}$ to $f_{27}$.

Function	Data type	BBPSO	DLSBBPSO	ETBBPSO	PBBPSO	TBBPSO	PBSO
21	Mean	1.100E+03	1.053E+03	1.114E+03	1.137E+03	1.101E+03	9.391E+02
	STD	1.548E+02	1.363E+02	1.593E+02	1.705E+02	1.750E+02	1.214E+02
	Best	6.060E−02	2.048E+00	6.230E+02	2.932E+01	2.250E+02	1.552E−01
	Worst	5.915E+04	5.297E+04	1.256E+05	5.966E+04	1.359E+05	9.351E+04
	Rank	3	2	5	6	4	1
22	Mean	2.698E+04	3.144E+04	2.599E+04	3.253E+04	2.575E+04	1.832E+04
	STD	8.068E+03	4.658E+03	8.305E+03	3.969E+03	6.335E+03	6.461E+03
	Best	6.060E−02	2.048E+00	6.230E+02	2.932E+01	2.250E+02	1.552E−01
	Worst	5.915E+04	5.297E+04	1.256E+05	5.966E+04	1.359E+05	9.351E+04
	Rank	4	5	3	6	2	1
23	Mean	1.275E+03	1.224E+03	1.286E+03	1.283E+03	1.308E+03	1.218E+03
	STD	1.282E+02	1.031E+02	1.200E+02	1.172E+02	1.376E+02	9.126E+01
	Best	6.060E−02	2.048E+00	6.230E+02	2.932E+01	2.250E+02	1.552E−01
	Worst	5.915E+04	5.297E+04	1.256E+05	5.966E+04	1.359E+05	9.351E+04
	Rank	3	2	5	4	6	1
24	Mean	1.883E+03	1.795E+03	1.876E+03	1.929E+03	1.904E+03	1.730E+03
	STD	1.911E+02	2.116E+02	1.766E+02	2.451E+02	1.565E+02	1.642E+02
	Best	6.060E−02	2.048E+00	6.230E+02	2.932E+01	2.250E+02	1.552E−01
	Worst	5.915E+04	5.297E+04	1.256E+05	5.966E+04	1.359E+05	9.351E+04
	Rank	4	2	3	6	5	1
25	Mean	7.627E+02	7.649E+02	7.713E+02	7.579E+02	7.438E+02	7.646E+02
	STD	6.553E+01	6.258E+01	5.340E+01	5.554E+01	7.415E+01	6.212E+01
	Best	6.060E−02	2.048E+00	6.230E+02	2.932E+01	2.250E+02	1.552E−01
	Worst	5.915E+04	5.297E+04	1.256E+05	5.966E+04	1.359E+05	9.351E+04
	Rank	3	5	6	2	1	4
26	Mean	1.404E+04	1.331E+04	1.458E+04	1.447E+04	1.503E+04	1.283E+04
	STD	1.429E+03	1.859E+03	1.929E+03	1.750E+03	1.771E+03	1.541E+03
	Best	6.060E−02	2.048E+00	6.230E+02	2.932E+01	2.250E+02	1.552E−01
	Worst	5.915E+04	5.297E+04	1.256E+05	5.966E+04	1.359E+05	9.351E+04
	Rank	3	2	5	4	6	1
27	Mean	5.000E+02	5.000E+02	5.000E+02	5.000E+02	5.000E+02	5.000E+02
	STD	5.429E−04	4.155E−04	4.961E−04	4.310E−04	3.557E−04	3.608E−04
	Best	6.060E−02	2.048E+00	6.230E+02	2.932E+01	2.250E+02	1.552E−01
	Worst	5.915E+04	5.297E+04	1.256E+05	5.966E+04	1.359E+05	9.351E+04
	Rank	2	5	3	6	4	1

**Table 10:** Simulation results of BBPSO, DLSBBPSO, ETBBPSO, PBBPSO, TBBPSO and PBSO, $f_{28}$ to $f_{29}$.

Function	Data type	BBPSO	DLSBBPSO	ETBBPSO	PBBPSO	TBBPSO	PBSO
28	Mean	5.000E+02	5.000E+02	5.000E+02	5.000E+02	5.000E+02	5.000E+02
	STD	5.926E−04	4.503E−04	4.921E−04	3.790E−04	3.394E−04	6.068E−04
	Best	6.060E−02	2.048E+00	6.230E+02	2.932E+01	2.250E+02	1.552E−01
	Worst	5.915E+04	5.297E+04	1.256E+05	5.966E+04	1.359E+05	9.351E+04
	Rank	2	5	3	6	4	1
29	Mean	4.552E+03	4.388E+03	4.217E+03	4.330E+03	4.500E+03	4.005E+03
	STD	9.481E+02	6.939E+02	7.639E+02	7.328E+02	8.772E+02	7.758E+02
	Best	6.060E−02	2.048E+00	6.230E+02	2.932E+01	2.250E+02	1.552E−01
	Worst	5.915E+04	5.297E+04	1.256E+05	5.966E+04	1.359E+05	9.351E+04
	Rank	6	4	2	3	5	1
Average Rank		3.45	3.69	3.82	4.55	3.97	1.52
Population Size: 100							
Max Iteration times: 10000							
Dimension: 100							
Search Range: [−100 100]							
Independent Runs: 37							

### Discussion

In both sets of experiments, PBSO consistently outperformed other methods. When compared to nature-inspired algorithms, PBSO achieved impressive results with 9 first-place rankings, 13 second-place rankings, 5 third-place rankings, 1 fourth-place ranking, and 1 fifth-place ranking. On average, it ranked 2.03, securing the top position among all algorithms. However, PBSO’s performance was less satisfactory when applied to single-modal test functions. This can be attributed to the fact that PBSO was not originally designed with a specialized evolutionary strategy for single-modal functions, which presents an important avenue for future research.

In contrast to PSO-based algorithms, PBSO excelled with 23 first-place rankings, 2 second-place rankings, 1 third-place ranking, 1 fourth-place ranking, and 1 sixth-place ranking, averaging an impressive 1.52 across all rankings and taking the first position among all algorithms. The experimental results suggest that, compared to traditional particle swarm algorithms, PBSO offers several advantages, including higher optimization accuracy, a simpler structure, and greater ease of understanding. More specifically, the pairwise barracuda structure significantly enhances interconnections between barracuda individuals, while the deep memory mechanism increases their chances of escaping local optima in high-dimensional search spaces. Furthermore, the leadership barracuda, equipped with a three-layer memory setting and a focus on balancing search resources, enhances the overall search accuracy of the entire barracuda group. In summary, the experiments clearly demonstrate that PBSO is capable of providing highly precise solutions for high-dimensional single-objective optimization problems.

## Conclusions

In this study, we introduce a novel metaheuristic approach inspired by nature, known as the Pair Barracuda Swarm Optimization algorithm (PBSO). PBSO is designed to emulate the social structure and collective behavior observed in barracuda swarms. The Pair Barracuda structure enhances the ability of individual barracudas to escape local optima. To enhance the search accuracy in high-dimensional spaces, we have devised an innovative iterative strategy. Notably, both the new structure and the iterative strategy have linear complexity, resulting in a time complexity of O(n) for PBSO. PBSO is compared to its predecessor, PBSO, and is found to be simpler, more user-friendly, and more robust in functional simulations. The experimental results consistently support PBSO’s superior performance. To further evaluate PBSO’s capabilities, we conducted high-dimensional simulations using the CEC2017 benchmark functions with a test dimension of 100. These experimental results firmly establish PBSO as the leading algorithm across all tested scenarios, providing dependable solutions for high-dimensional optimization challenges. However, it’s worth noting that PBSO tends to converge towards local optima when dealing with combinatorial optimization problems. This issue is attributed to the limited information transfer from the barracuda leader and the shallow memory of the barracuda pair. Consequently, future research should focus on improving the speed of information transfer from the barracuda leader to the common barracuda and enhancing the memory depth of barracudas. Additionally, exploring the application of PBSO in real-world scenarios, such as wireless sensor networks, holds promise for future investigations.

## Data Availability

The datasets used and/or analysed during the current study available from the corresponding author on reasonable request.
